# Arylic C–X Bond Activation by Palladium Catalysts: Activation Strain Analyses of Reactivity Trends

**DOI:** 10.1038/s41598-018-28998-3

**Published:** 2018-07-16

**Authors:** Pascal Vermeeren, Xiaobo Sun, F. Matthias Bickelhaupt

**Affiliations:** 1 Department of Theoretical Chemistry and Amsterdam Center for Multiscale Modeling, Vrije Universiteit Amsterdam, De Boelelaan 1083, 1081 HV Amsterdam, The Netherlands; 2 Radboud University Nijmegen, Institute for Molecules and Materials, Heyendaalseweg 135, 6525 AJ Nijmegen, The Netherlands

## Abstract

We have quantum chemically explored arylic carbon–substituent bond activation via oxidative insertion of a palladium catalyst in C_6_H_5_X + PdL_n_ model systems (X = H, Cl, CH_3_; L_n_ = no ligand, PH_3_, (PH_3_)_2_, PH_2_C_2_H_4_PH_2_) using relativistic density functional theory at ZORA-BLYP/TZ2P. Besides exploring reactivity trends and comparing them to aliphatic C–X activation, we aim at uncovering the physical factors behind the activity and selectivity. Our results show that barriers for arylic C–X activation are lower than those for the corresponding aliphatic C–X bonds. However, trends along bonds or upon variation of ligands are similar. Thus, bond activation barriers increase along C–Cl < C–H < C–C and along Pd < Pd(PH_3_) or Pd(PH_2_C_2_H_4_PH_2_) < Pd(PH_3_)_2_. Activation strain analyses in conjunction with quantitative molecular orbital theory trace these trends to the rigidity and bonding capability of the various C–X bonds, model catalysts, and ligands.

## Introduction

**Figure 1:**
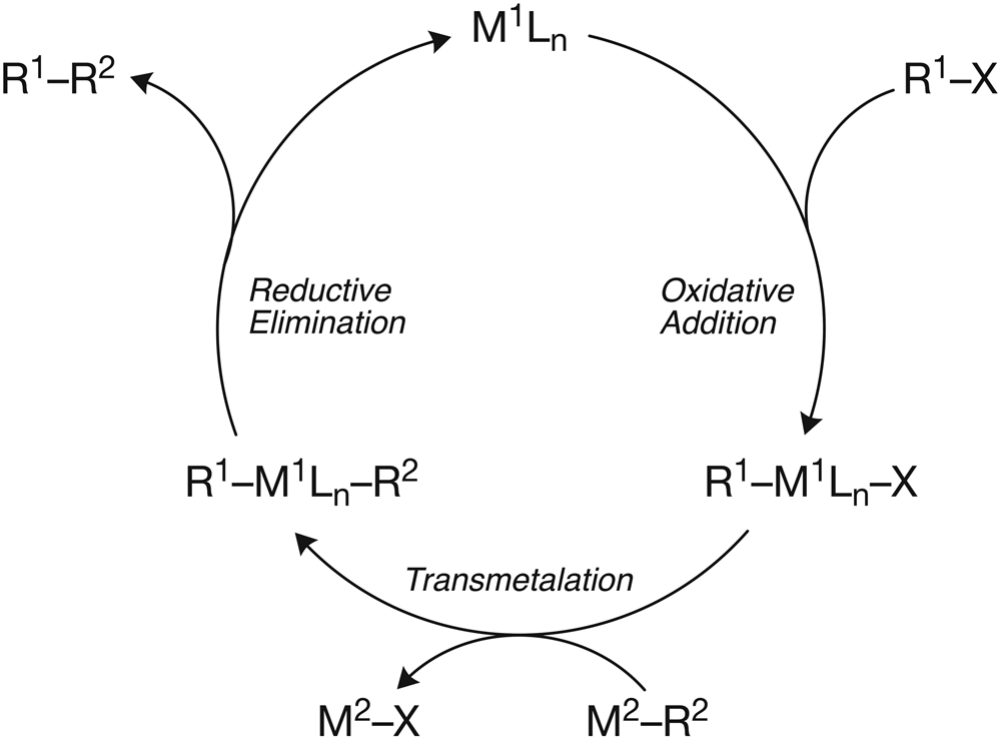
Generic catalytic cycle of a transition-metal-catalyzed cross-coupling reaction.

Catalysis plays a key role in many industrial processes, as well as in the synthesis of various biologically active compounds^1–3^. An important class of catalytic processes is constituted by transition-metal-catalyzed cross-coupling reactions which furnish new carbon–carbon bonds (Fig. 1)^4,5^. The first and commonly the rate-determining step in the catalytic cycle of a typical cross-coupling reaction is the activation of a carbon–substituent bond (R–X) by oxidative addition to a transition metal complex. This reaction step plays an essential role in the selectivity and efficiency of the overall catalytic cycle. The oxidative addition can in principle proceed via various reaction mechanisms, for instance, the concerted pathway. This pathway is an associative substitution reaction in which R–X binds first as a σ complex and then undergoes R–X bond breaking as a result of strong back donation from the metal d orbitals into the σ*_R–X_ orbital. Another possible pathway proceeds analogously to the well-known bimolecular nucleophilic substitution. A direct nucleophilic attack of the metal center at the more electropositive atom of the R–X bond by donating the catalyst’s d electrons into the substrate’s σ* orbital, forming an [M–R]^+^ species and an X^−^ leaving group.

Due to the importance of the oxidative addition, extensive experimental and theoretical research has been committed to this reaction step^6–19^. Earlier theoretical studies have investigated the oxidative insertion of numerous d^10^ transition-metal catalysts into various aliphatic C–X bonds^6,8,16,17^. Additionally, several concepts were introduced on how bond-activation barriers of d^10^-metal complexes depend on and can be tweaked through, the electronic and steric properties of the metal center and the ligands of the catalyst complex^16,17^. Furthermore, previous studies have shown that solvation does not alter the trends in oxidative-insertion reactivity and selectivity along various aliphatic bonds and model catalysts compared to the gas phase^10,17,19^.

**Figure 2:**
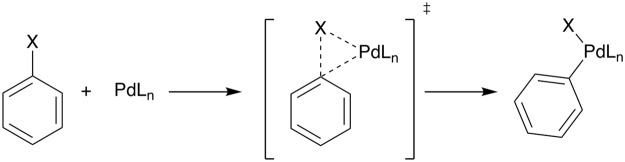
General representation of the arylic C–X bond activation by a palladium catalyst, where X = H, Cl or CH_3_; L_n_ = no ligand, PH_3_, (PH_3_)_2_, PH_2_C_2_H_4_PH_2_.

Herein, we quantum chemically explore and analyze reactivity and selectivity trends in arylic carbon–substituent bond activation of C–H, C–Cl, and C–C bonds in benzene, chlorobenzene, and toluene to palladium catalysts Pd, Pd(PH_3_), Pd(PH_3_)_2_, and Pd(PH_2_C_2_H_4_PH_2_) (Fig. 2). Our computations have been performed using relativistic density functional theory (DFT) at ZORA-BLYP/TZ2P level of theory^20–37^, as implemented in the Amsterdam Density Functional (ADF) program. Reactivity trends have been analyzed using the activation strain model (ASM) in conjunction with quantitative canonical (Kohn-Sham) molecular orbital theory and a matching canonical energy decomposition analysis (EDA) that quantifies the various features in the bonding mechanism. First, we compare the behavior and trends found for the arylic C–X activation with the previously studied aliphatic C–X bonds^16,17^. In the second part, we examine the activation of the C–H bond of benzene, the C–Cl bond of chlorobenzene, and the exocyclic C–C bond of toluene by a bare palladium atom. At last, we investigate the effect of introducing various phosphine ligands on the different reaction pathways.

## Results and Discussion

### Reaction Profiles

**Figure 3:**
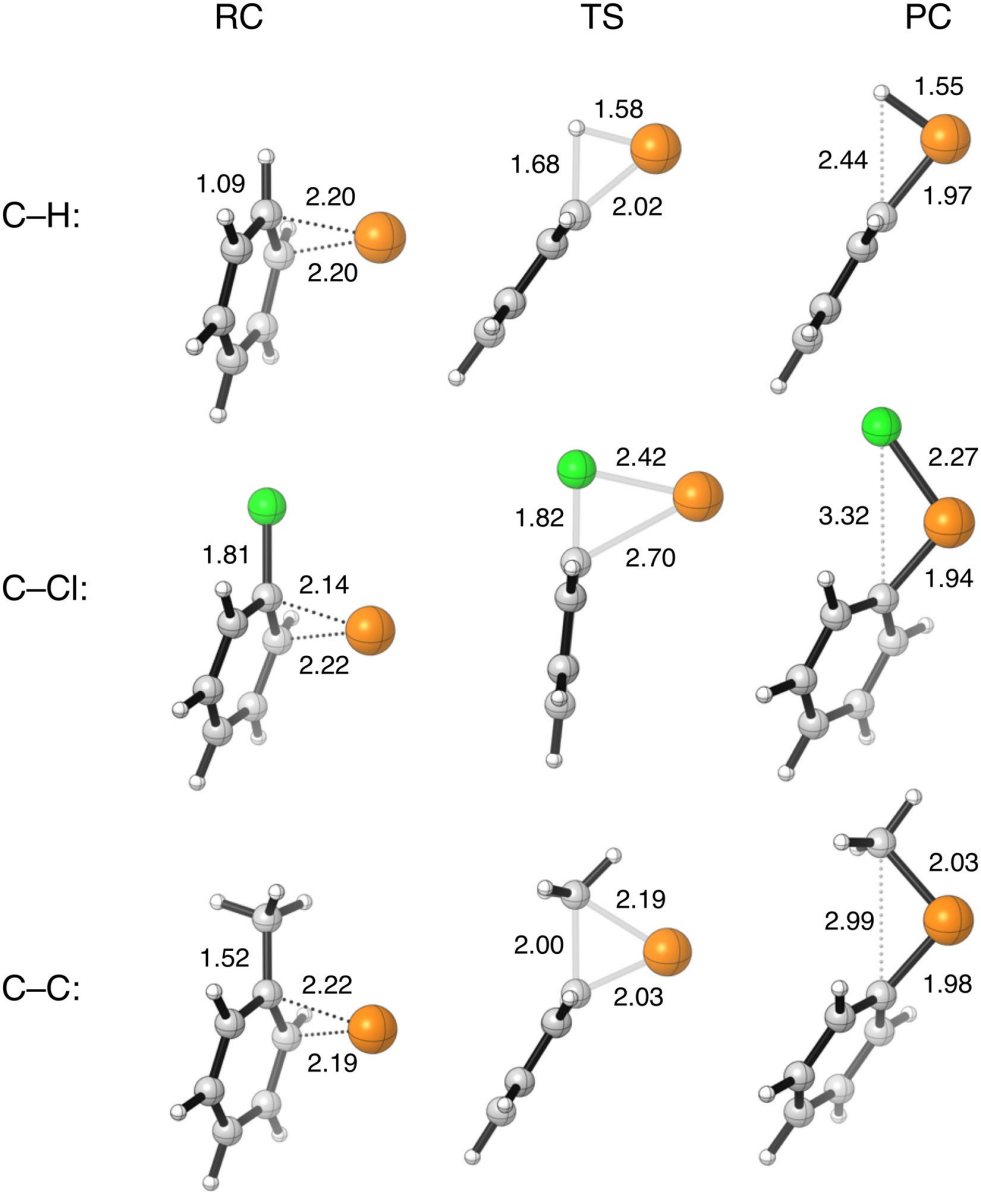
Representative geometries of the stationary points (in Å) for the oxidative insertion of Pd into the benzene C–H, chlorobenzene C–Cl, and toluene C–C bond, computed at ZORA-BLYP/TZ2P.

**Figure 4:**
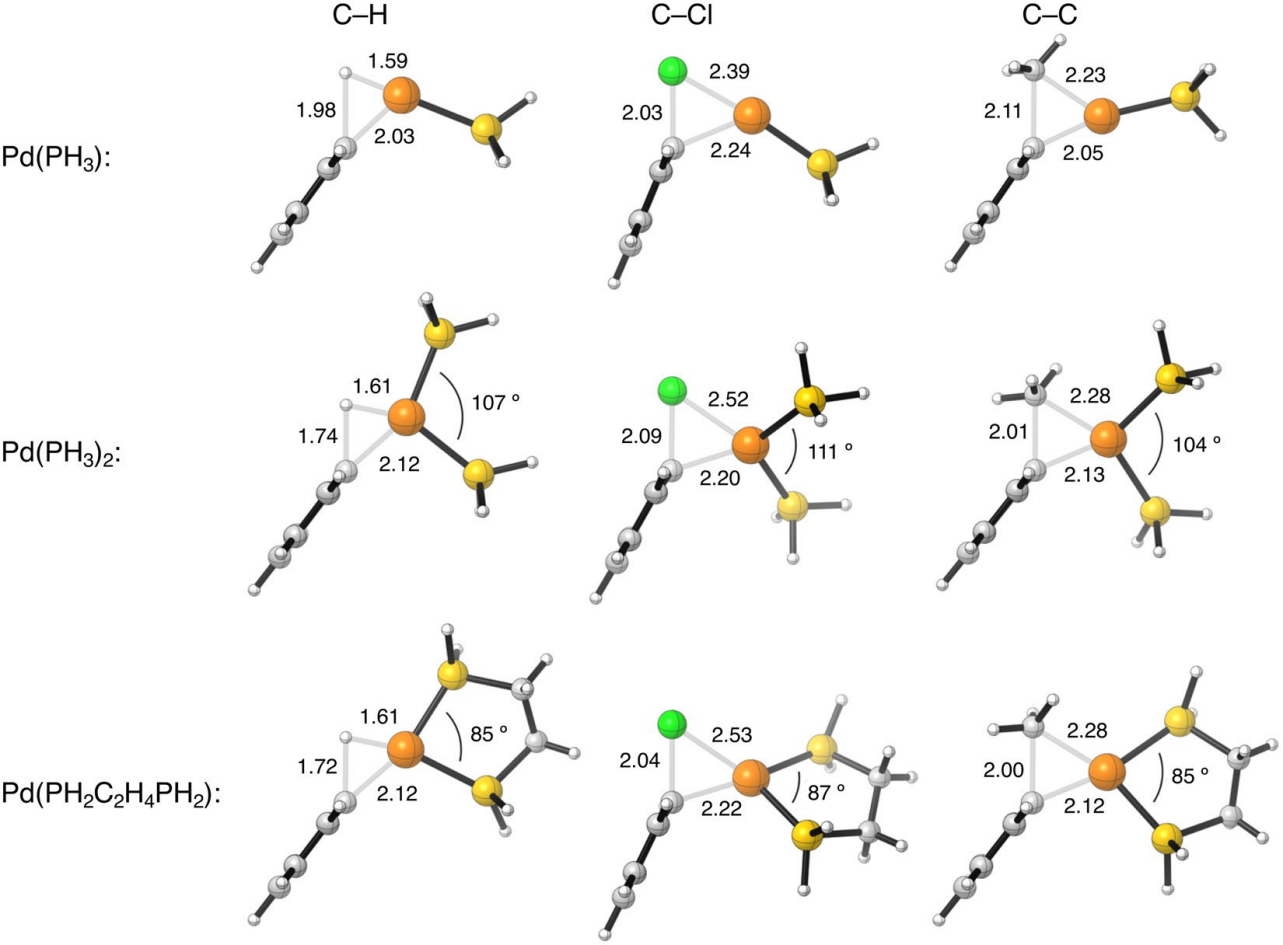
Representative geometries of the transition states (in Å, deg) of the oxidative insertion of Pd(PH_3_), Pd(PH_3_)_2_, and Pd(PH_2_C_2_H_4_PH_2_) into the C–H, C–Cl, and C–C bond of benzene, chlorobenzene, and toluene, computed at ZORA-BLYP/TZ2P.

Our ZORA-BLYP/TZ2P results are collected in Table 1 (relative energies) and Figs 3 and 4 (structural data); for enthalpies and more detailed structural information, see the Supplementary Information. A number of general trends can be observed. In the first place, the barriers for arylic C–X bond activation in Table 1 are 4–9 kcal mol^−1^ lower than those computed earlier. for the corresponding aliphatic C–X bonds^16,17,19^. An exception is Pd(PH_3_)-induced C–H bond activation. In that case, arylic C–H activation proceeds via a slightly *higher* barrier than aliphatic (methane) C–H activation, 17.1 and 15.7 kcal mol^−1^, respectively. Secondly, the trends in reaction barrier, for a given palladium catalyst, along the three C–X bonds is similar for the arylic and aliphatic C–X bonds and decreases in the order C–C > C–H > C–Cl^6,8,16,17^. Thus, the activation of the ethane and toluene C–C bonds by a palladium catalyst occur with the highest barriers, contrarily, the activation of the chloromethane and chlorobenzene C–Cl bonds have the lowest barriers.

**Table 1:** Electronic energies^[a]^ of stationary points relative to separate reactants (in kcal mol^−1^) for the oxidative addition of arylic (C_6_H_5_X)^[b]^ and aliphatic (CH_3_X)^[c]^ C–H, C–Cl, and C–C bonds to various model catalysts.

C–X bond	Model catalyst	RC		TS		PC	
		C_6_H_5_X	(CH_3_X)	C_6_H_5_X	(CH_3_X)	C_6_H_5_X	(CH_3_X)
C–H	Pd	−21.7	(−6.7)	−1.6	(4.0)	−6.0	(−3.6)
	Pd(PH_3_)	−14.9	(−7.7)	17.1	(15.7)	16.9	(14.9)
	Pd(PH_3_)_2_	−0.4	(0.0)	27.4	(32.6)	24.2	(27.3)
	Pd(PH_2_C_2_H_4_PH_2_)	−7.7	(−1.3)	13.6	(18.6)	10.0	(12.6)
C–Cl	Pd	−19.2	(−12.9)	−8.1	(−0.6)	−33.8	(−33.5)
	Pd(PH_3_)	−14.4	(−12.9)	−4.4	(2.3)	−23.8	(−26.9)
	Pd(PH_3_)_2_	−0.4	(−0.5)	19.2	(27.1)	−9.6	(−11.6)
	Pd(PH_2_C_2_H_4_PH_2_)	−8.8	(−6.4)	5.5	(14.3)	−24.9	(−27.1)
C–C	Pd	−19.7	(−6.7)	11.0	(18.5)	−8.5	(−9.4)
	Pd(PH_3_)	−15.1	(−7.9)	22.8	(26.3)	14.6	(13.6)
	Pd(PH_3_)_2_	−0.3	(0.0)	45.7	(51.7)	27.1	(26.6)
	Pd(PH_2_C_2_H_4_PH_2_)	−5.3	(−1.7)	33.7	(38.4)	12.0	(11.3)

[a] Computed at ZORA-BLYP/TZ2P. [b] This work. [c] From refs^16,17,19^.

Furthermore, we have examined the effect of various phosphine ligands on the reactivity of the catalyst, by changing the catalyst from bare palladium to a palladium metal center with ligands varying from one phosphine (PH_3_), to two phosphines ((PH_3_)_2_), to the chelating 1,2-ethanediyldiphosphine (PH_2_C_2_H_4_PH_2_). These phosphine ligands have both σ-donor and π-acceptor properties. Coordinating these ligands to the catalyst increases the reaction barriers of all arylic C–X bond activations (Fig. 4). This is again similar to the trend in reaction barriers that was computed for the oxidative addition of aliphatic C–X bonds to palladium-phosphine catalysts^16,17^. The reaction barriers of the arylic C–H bond activation, for example, increase from −1.6 to 17.1 to 27.4 kcal mol^−1^ when the catalyst changes along a bare Pd atom, Pd(PH_3_), and Pd(PH_3_)_2_. However, when we coordinate a chelating ligand to the catalyst, i.e., Pd(PH_2_C_2_H_4_PH_2_), the reaction barrier drops to 13.6 kcal mol^−1^, that is, below the barriers for Pd(PH_3_) and Pd(PH_3_)_2_. In the case of C–Cl and C–C bond activation, the barriers for the chelate complex Pd(PH_2_C_2_H_4_PH_2_) also drop below the one of Pd(PH_3_)_2_ but not below that for Pd(PH_3_). Thus, the trend in barriers is: Pd < Pd(PH_3_) or Pd(PH_2_C_2_H_4_PH_2_) < Pd(PH_3_)_2_.

The reactant complexes (RC) for arylic C–H, C–Cl, and C–C activation by a bare Pd atom are all more stable than their aliphatic counterparts (Table 1). The higher stability of the arylic reactant complexes originates from the relatively favorable *η*^2^ interaction of the Pd center with the substrate’s aromatic π system at C1 and C2. The aliphatic reactant complexes feature a weaker palladium–substrate interaction: *η*^2^ with two C–H bonds in the case of methane and ethane, and *η*^1^ with the chlorine lone pair of the C–Cl bond of chloromethane. Moreover, the products of all bond activations by a bare Pd atom are stable relative to the reactants. The most stable product is found for the activation of the C–Cl bond, followed by C–C and C–H bond activation.

The introduction of ligands coordinated to the palladium catalyst has the effect of weakening reactant complexes and pushing up reaction barriers (Table 1). In the case of chlorobenzene C–Cl activation, for instance, coordinating one PH_3_ ligand to Pd destabilizes the RC by 5 kcal mol^−1^, whereas, adding a second phosphine ligand reduces this RC’s stability with another 14 kcal mol^−1^. The substantial reduction of RC stability, when going from a monoligated to a bisligated palladium-phosphine catalyst, is due to the loss of the energetically favorable *η*^2^ interaction between the catalyst and the phenyl ring of the substrate as the steric crowding around the metal center increases. Going from Pd(PH_3_)_2_ to Pd(PH_2_C_2_H_4_PH_2_), the RC becomes more stable as there is again sufficient room for entering into a favorable *η*^2^ interaction between palladium and the substrate’s π system at C1 and C2. A similar trend in reactant complex stability is found for the activation of the arylic C–H and C–C bonds.

### Activation Strain Analyses of Aliphatic C–X Versus Arylic C–X Bond Activation

To analyze and compare the reaction pathways of the aliphatic and arylic C–X bond activation by a bare Pd atom catalyst, we applied the activation strain model^38–40^. This model is based on the idea that the energy of a reacting system, that is, the reaction potential energy surface, is described with respect to, and understood in terms of the properties of, the original reactants. It considers their rigidity and the extent to which the reactants must deform during the reaction plus their capability to interact as the reaction proceeds. Thus, we decompose the total energy, Δ*E*(ζ), into the strain and interaction energy, i.e., Δ*E*_strain_(ζ) and Δ*E*_int_(ζ), and projected these values onto the bond stretch of the activated C–X bond (equation 1).

1 $${\rm{\Delta }}E(\zeta )={\rm{\Delta }}{E}_{{\rm{strain}}}(\zeta )+{\rm{\Delta }}{E}_{{\rm{int}}}(\zeta )$$

In this equation, the strain energy Δ*E*_strain_(ζ) is the energy that is necessary to deform the reactants from their equilibrium structure to the geometry they adopt in the reaction system at point ζ of the reaction coordinate. On the other hand, the interaction energy Δ*E*_int_(ζ) accounts for all the chemical interactions that occur between the deformed fragments during the reaction.

**Figure 5:**
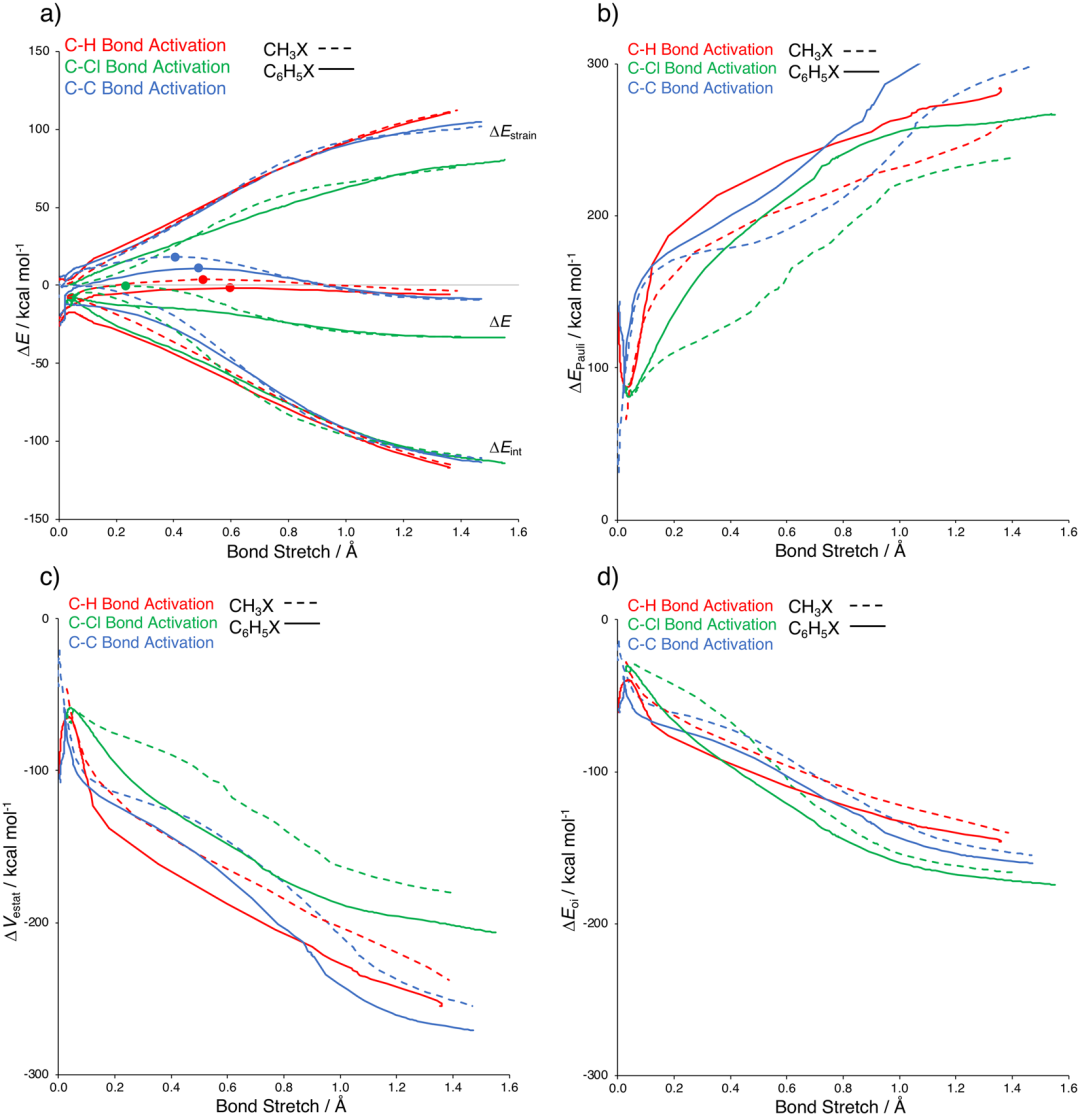
(**a**) Activation strain diagram comparing the oxidative addition of the aliphatic C–X bond versus the arylic C–X bond (X = H, Cl, CH_3_) by a Pd catalyst, where the transition states are indicated with a dot. Energy decomposition analysis terms of the oxidative addition of the aliphatic C–X bond versus the arylic C–X bond (X = H, Cl, CH_3_) by a Pd catalyst: (**b**) Pauli repulsion; (**c**) electrostatic interaction; and (**d**) orbital interaction. Computed at ZORA-BLYP/TZ2P.

Our activation strain analyses reveal that the higher reaction barrier of the activation of the aliphatic C–X bond (see Table 1) appears to be caused by an initially less stabilizing interaction energy compared to the situation for arylic C–C bond activation (see Fig. 5a; zoomed-in version see Supplementary Figure 4a). The strain energies do not discriminate: they are identical for the activation of aliphatic and arylic C–X bonds with equal substituents. Proceeding further along the reaction coordinate the difference in interaction energy between the two reaction systems diminishes and eventually we end up with comparable total energies at the product side. In other words, reaction energies for aliphatic and arylic C–X activation are similar.

Note that this circumstance, i.e., more stabilizing interactions for arylic than for aliphatic C–X activation at the beginning of the reaction and not so different interactions at the end, also makes the ∆*E*_int_ curves shallower for arylic C–X activation. Therefore, from aliphatic to arylic C–X activation, the TS shifts slightly to a later stage of the reaction, that is, it becomes more product like (see Fig. 5a). Only the already relatively product-like TS for C–Cl activation shifts to a yet earlier point along the reaction coordinate if we go from aliphatic to arylic C–X activation. The reason is a subtly steeper rise of the arylic C–X strain curve in the very early reaction stage (see Supplementary Figure 4b for a zoomed-in version).

To clarify the difference in interaction energy, we have further decomposed Δ*E*_int_ into three different terms using our canonical energy decomposition analysis (EDA) scheme^41–44^:

2 $${\rm{\Delta }}{E}_{{\rm{int}}}(\zeta )={\rm{\Delta }}{V}_{{\rm{elstat}}}(\zeta )+{\rm{\Delta }}{E}_{{\rm{Pauli}}}(\zeta )+{\rm{\Delta }}{E}_{{\rm{oi}}}(\zeta )$$

Herein, Δ*V*_elstat_ is the classical electrostatic interaction between the unperturbed charge distributions of the (deformed) reactants which is usually attractive. The Pauli repulsion Δ*E*_Pauli_ comprises the destabilizing interaction between occupied orbitals (more precisely, between electrons of like spin) due to the Pauli principle and is responsible for any steric repulsion. The orbital interaction energy Δ*E*_oi_ accounts for polarization and charge transfer, amongst others, HOMO–LUMO interactions.

Our energy decomposition analyses show that both the orbital interactions ∆*E*_oi_ and the electrostatic attraction ∆*V*_elstat_ are more stabilizing for the arylic C–X activation processes (see Fig. 5b–d). It is their combined action that overrules the trend of the somewhat more destabilizing steric (Pauli) repulsion and makes the net interaction ∆*E*_int_ systematically more *stabilizing* for the arylic than for the aliphatic C–X activation.

The characteristic difference in orbital interactions at the beginning of the oxidative addition reactions (i.e., the fact that they are less stabilizing for aliphatic than for arylic C–X activation) can be ascribed to differences in the bonding mechanisms between the catalyst and substrate along the reaction coordinate. The catalyst–substrate interaction for all three aliphatic reactant complexes are dominated by the donor–acceptor interaction between the filled σ_C–X_ orbital of the substrate (or LP_Cl_) in early stages of C–X activation and the empty 5 s orbital of the palladium catalyst, associated with HOMO–LUMO energy gaps between 5.5 and 3.5 eV. When the reaction proceeds further towards the transition state, the backbonding interaction between the catalyst 4d and substrate σ*_C–X_ orbital becomes the most important mechanism.

At variance, for the arylic reactant complexes, the foremost donor–acceptor interaction in the reactant complex is between the filled palladium 4d orbitals and the low lying empty π* orbital of the phenyl ring, which goes with a smaller HOMO–LUMO gap of 2.7–3.3 eV and thus a more stabilizing orbital interaction. Proceeding further along the reaction coordinate, the bonding mechanism changes, from solely metal–substrate 4d → π* “backbonding” to a “forward” donation from the occupied aromatic π orbitals into the empty metal 5s.

As soon as the reaction approaches the transition state, the orbital-interaction mechanism becomes, in all cases, predominantly backbonding from catalyst 4d to substrate σ*_C–X_ orbital, just as in the case of the aliphatic C–X activation. Importantly, the σ*_C–X_ acceptor orbital of the aliphatic C–X bond is at higher energy than that of the arylic C–X bond. Consequently, the HOMO–LUMO gap associated with the metal 4d to substrate σ*_C–X_ interaction is larger in the case of the aliphatic C–X bond which therefore experiences a less stabilizing catalyst–substrate interaction and thus arrives at a higher barrier in the bond activation reaction.

### Variation along C–C, C–H, and C–Cl Bonds

Our activation strain analyses clearly reveal the physical factors that determine the trend of a decreasing barrier along the activation of arylic C–C, C–H, and C–Cl (see Fig. 5a). These trends and the physical mechanisms behind them are very similar to those found for the aliphatic C–X bond activation reactions (see Table 1)^6,8,16,17^. The low barrier for C–Cl activation is simply the result of the relative weakness of this bond. We compute homolytic bond dissociation energies for our arylic C–C and C–H bonds (132.3 and 143.7 kcal mol^−1^) that are significantly higher than that for the arylic C–Cl bond (only 106.9 kcal mol^−1^). This results in a significantly lower strain curve and thus a lower barrier for the activation of the arylic C–Cl bond.

**Figure 6:**
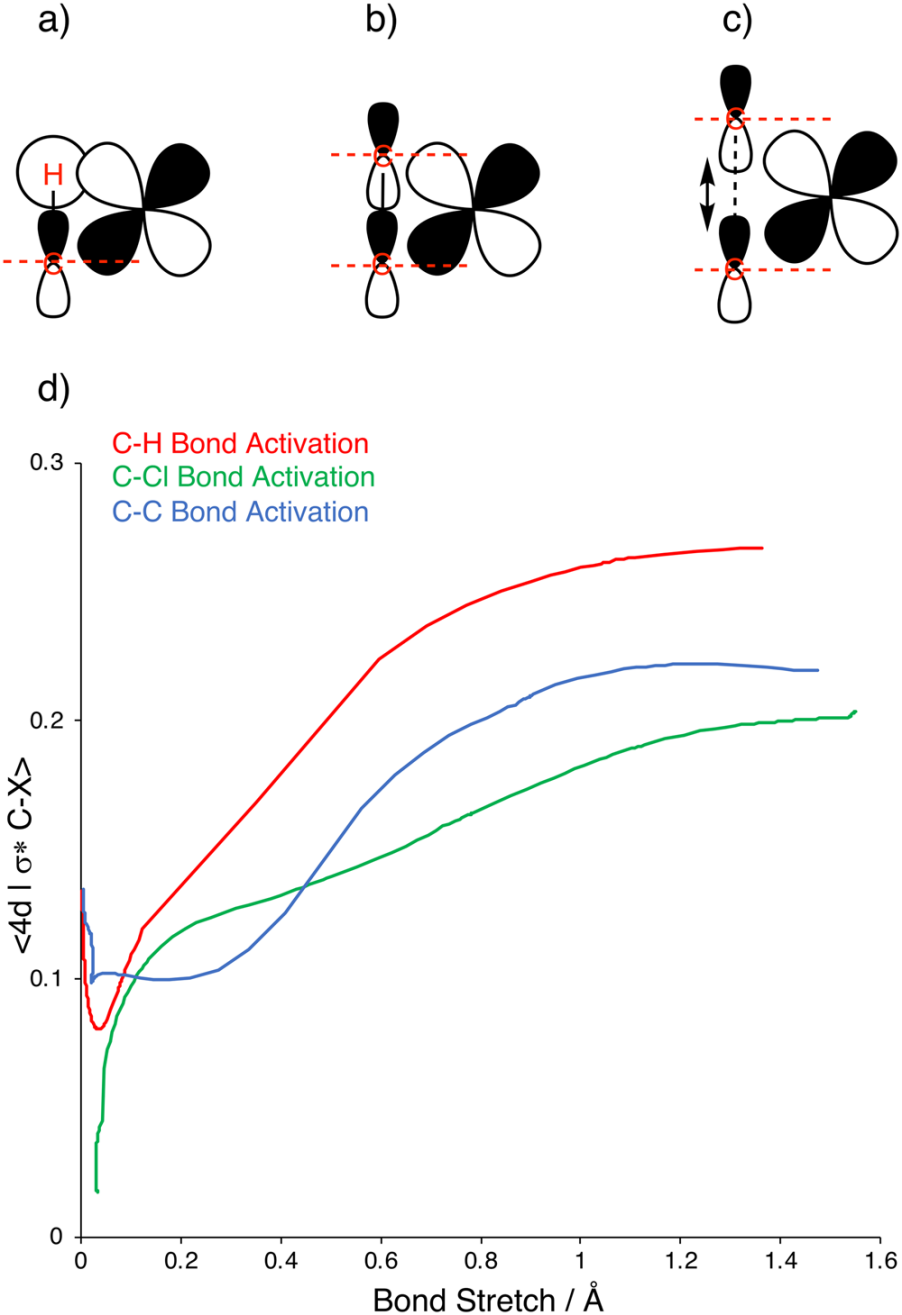
Schematic representation of the overlap of the filled transition-metal d orbital with the σ* orbital of (**a**) a C–H bond; (**b**) a C–C bond; (**c**) an elongated C–C bond (dashed red lines represent the nodal plane of the carbon 2p atomic orbital); and (**d**) overlap integral of the backbonding interaction between Pd 4d_π_ and the arylic C–X antibonding σ*_C–X_ orbitals along Pd + C_6_H_5_–X oxidative addition reactions, computed at ZORA-BLYP/TZ2P.

The fact that arylic C–C activation goes with a higher barrier than arylic C–H activation can be traced to a delay, along the reaction coordinate, in building up stabilizing interaction ∆*E*_int_. The physical origin consists of the same two factors as those found for the corresponding aliphatic C–H and C–C bonds^6,8,16,17^: (i) the methyl C–H bonds shield the C–C bond which first has to stretch before the metal can approach sufficiently closely for its d_π_ orbital to overlap with the σ*_C–C_; and (ii) the C–C bond also has to stretch in order to move the two nodal surfaces that the σ*_C–C_ orbital has on the carbon nuclei, out of the way of the metal d_π_ lobes before a favorable 〈d_π_|σ*_C–C_〉 overlap can occur. This is schematically illustrated in Fig. 6a–c whereas the quantitative course of the overlap of the 〈d_π_|σ*_C–X_〉 overlaps is shown in Fig. 6d. Note, in the latter, the more continuous increase of 〈d_π_|σ*_C–H_〉 overlap as opposed to the more abrupt rise of 〈d_π_|σ*_C–C_> after a stretch of approximately 0.3 Å.

### Variation along Model Catalysts Pd, Pd(PH_3_), Pd(PH_3_)_2_, and Pd(PH_2_C_2_H_4_PH_2_)

**Figure 7:**
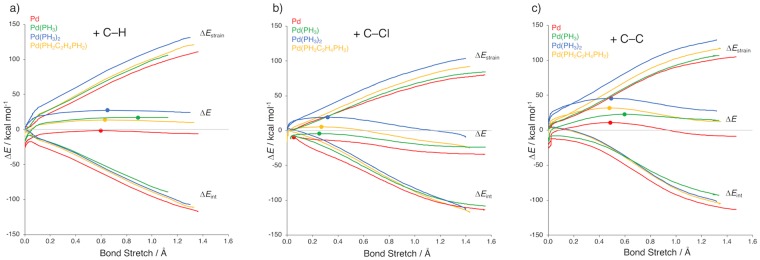
Activation strain diagram comparing the insertion of different catalysts into the (**a**) benzene C–H; (**b**) chlorobenzene C–Cl; and (**c**) toluene C–C, where the transition states are indicated with a dot. Computed at ZORA-BLYP/TZ2P.

Finally, we examine how the activity towards activating a particular C–X bond depends on the choice of ligands, by varying the model catalyst along Pd, Pd(PH_3_), Pd(PH_3_)_2_, and Pd(PH_2_C_2_H_4_PH_2_) (see Fig. 7). The principal effect of introducing phosphine ligands is a raise of all C–X activation barriers, with major differences between the various ligands as detailed below. The selectivity for the three different bonds, however, remains identical, that is, barriers decrease in the order C–C > C–H > C–Cl for each of our model catalysts.

A more careful inspection of reactivity trends shows that C–X activation barriers increase along Pd, Pd(PH_3_), and Pd(PH_3_)_2_, and then decrease again from Pd(PH_3_)_2_ to Pd(PH_2_C_2_H_4_PH_2_) (see Table 1). These kinetic tendencies are mirrored by analogous trends in the reaction energies. These findings are reminiscent of the behavior reported earlier for aliphatic C–X activation^11,16,17^. Our activation strain analyses reveal a clear set of physical mechanisms behind these reactivity trends (see Fig. 7). Introducing one phosphine ligand stabilizes the palladium d orbitals which leads to a weaker HOMO–LUMO and overall interaction curve ∆*E*_int_ and, thus to a higher total energy profile ∆*E*. Note that the strain curve is not much affected yet.

That changes as soon as the second phosphine ligand is introduced: In the linear d^10^-Pd(PH_3_)_2_ complex, the palladium center has become sterically more congested. Now, it has to bend its two ligands away by up to approximately 100° to avoid steric (Pauli) repulsion with, in order to make room for, the incoming substrate. This bending goes with a substantial enhancement of the unfavorable strain curve ∆*E*_strain_ which further raises the reaction barrier provided by the total energy profile ∆*E* (see Fig. 7) Note that bending is also required to achieve a good overlap between the catalyst hybrid d and the substrate σ*_C–X_ orbital (*vide supra*).

In Pd(PH_2_C_2_H_4_PH_2_), the coordinating sites are pulled towards each other by the short dimethylene bridge which causes the catalyst complex to be pre-distorted. Consequently, substantially less additional bending is required for coordinating the substrate. This has a major effect on the strain curve ∆*E*_strain_ which drops markedly (see Fig. 7). The effect on the interaction curve is much less pronounced. Therefore, the barrier lowering and thus rate enhancement that goes with chelate ligands and smaller bite angles in catalyst complexes mainly originate from the reduction of catalyst bending strain.

Finally, we wish to point out an interesting detail. Going from the monoligated to the bisligated catalyst, one can observe anti-Hammond behavior (see Fig. 7a,c)^16^. Thus, C–H and C–C bond activation by Pd(PH_3_) proceeds with a lower activation barrier than Pd(PH_3_)_2_. Yet, the TS of the former more exothermic reaction is located a later, more product-like point along the reaction coordinate, instead of at an earlier, more reactant-like point. The reason is that the electron-donating capability of the bisligated catalyst suddenly improves, relative to that of Pd(PH_3_), only after the P–Pd–P angle starts bending. The effect of this phenomenon is that the resulting hybrid d orbitals are pushed up in energy and oriented more towards the substrate σ*_C–X_ orbital, resulting in a better overlap. Thus, as we proceed along the reaction coordinate for oxidative addition to the bisligated metal complex, the interaction curve becomes steeper. This enhanced steepness pulls the TS ‘to the left’, that is, to an earlier reactant-like geometry.

## Conclusion

We find that arylic C–X bond activation proceeds consistently via lower reaction barriers than the corresponding processes for aliphatic C–X bonds. However, trends along bonds or upon variation of ligands are similar. Thus, bond activation barriers increase along C–Cl < C–H < C–C and also along Pd < Pd(PH_3_) or Pd(PH_2_C_2_H_4_PH_2_) < Pd(PH_3_)_2_. This follows from our quantum chemical exploration of arylic carbon–substituent bond activation via oxidative insertion of a palladium catalyst in C_6_H_5_X + PdL_n_ model systems (X = H, Cl, CH_3_; L_n_ = no ligand, PH_3_, (PH_3_)_2_, PH_2_C_2_H_4_PH_2_) using relativistic density functional theory.

Activation strain analyses show that the lower reaction barrier for arylic as compared to aliphatic C–X bond activation is caused by a stronger, more stabilizing catalyst–substrate interaction for the former which can be traced back from more stabilizing orbital interactions assisted by stronger electrostatic attraction. The more stabilizing orbital interactions originate from a smaller HOMO–LUMO gap because arylic σ*_C–X_ orbitals are in general at lower energy than their aliphatic counterpart.

The high reaction barrier for both arylic and aliphatic C–C activation is caused by a delay in building up favorable catalyst–substrate interaction in early stages of the reaction. This delay in stabilizing interaction originates from the cancelation of metal d_π_–substrate σ*_C–X_ orbital overlap with the additional 2p atomic nodal surface in σ*_C–X_ when X = CH_3_. This unfavorable situation is only overcome after the reaction has proceeded already to some extent and the C–C bond has undergone sufficient stretching to allow for a better d_π_–σ*_C–C_ phase match. Coordination of phosphine ligands to the catalyst raises the barrier because of the additional catalyst strain that occurs upon bending ligands away to accommodate the substrate.

## Methods

All calculations are based on Density Functional Theory (DFT)^20,21^, and have been performed using the Amsterdam Density Functional (ADF) software^22–24^. The numerical integration is executed with Becke’s fuzzy cell integration scheme^25^. The molecular orbitals (MO) are expanded in a large uncontracted set of Slater-type orbitals (STOs). The used basis set, denoted TZ2P, is of triple-ζ quality for all atoms and has been improved by two sets of polarization functions^26^. The polarization functions are 2p and 3d on H, 3d and 4 f on C, P, Cl, and 5 p and 4 f on Pd. The molecular density was fitted by the Zlm fitting scheme^27^. The frozen core approximation has been employed using the following frozen shells: 1 s for C, [He]2p for Cl, and [Ar]3d for Pd.

Equilibrium structures and transition state geometries have been computed using analytical gradient techniques and the BLYP functional^28^. In the latter, the exchange is described by Slater’s Xα potential with nonlocal corrections due to Becke added self-consistently^29–31^, whereas correlation is treated using the gradient-corrected functional of Lee, Yang, and Parr^32^, added again in a self-consistent fashion. Scalar relativistic effects are accounted for using the zeroth-order regular approximation (ZORA)^33,34^. This approach was extensively tested against ab initio reference benchmarks from hierarchical series up till CCSD(T)^7,9,35–37^.

All stationary points have been verified, through vibrational analysis^45–47^, to be (local) minima (zero imaginary frequencies) or transition states (one imaginary frequency). The character of the normal mode associated with the imaginary frequency has been analyzed to ensure it resembles the reaction under consideration. To obtain the potential energy surface (PES) of the chemical process, intrinsic reaction coordinate (IRC) calculations have been performed^48,49^. The potential energy surfaces are analyzed using the PyFrag program^50^. Optimized structures were illustrated using CYLview^51^.

## Electronic supplementary material

Supplementary Information
