# Supplementary material for: Arylic C–X Bond Activation by Palladium Catalysts: Activation Strain Analyses of Reactivity Trends
Source: Sci Rep. 2018 Jul 16;8:10729. doi: 10.1038/s41598-018-28998-3 (PMC6048108; doi:10.1038/s41598-018-28998-3)
Supplement: Supplementary file 1 — Supplementary Information [file 41598_2018_28998_MOESM1_ESM.pdf]

Supplementary Information for

Arylic C–X Bond Activation by Palladium Catalysts: Activation Strain Analyses of Reactivity Trends

Pascal Vermeeren[1], Xiaobo Sun[1], and F. Matthias Bickelhaupt*[1,2]

1 Department of Theoretical Chemistry and Amsterdam Center for Multiscale Modeling, Vrije Universiteit Amsterdam, De Boelelaan 1083, 1081 HV Amsterdam, The Netherlands.

2 Radboud University Nijmegen, Institute for Molecules and Materials, Heyendaalseweg 135, 6525 AJ Nijmegen, The Netherlands.

E-mail: F.M.Bickelhaupt@vu.nl

Supplementary Table 1. Relative enthalpies (in kcal mol^{-1}) of stationary points along the oxidative addition of the benzene C–H, chlorobenzene C–Cl, and toluene C–C bond to the palladium–phosphine catalysts. [a]

		RC	TS	PC
C–H	Pd	-20.6	-3.8	-7.2
	$Pd(PH_3)$	-14.1	14.3	14.9
	$Pd(PH_3)_2$	-0.4	24.6	22.6
	$Pd(PH_2C_2H_4PH_2)$	-7.0	11.3	8.7
C–Cl	Pd	-10.6	-7.2	-33.1
	$Pd(PH_3)$	-10.8	-4.5	-22.8
	$Pd(PH_3)_2$	-0.5	18.5	-8.4
	$Pd(PH_2C_2H_4PH_2)$	-7.9	5.3	-23.3
C–C	Pd	-18.0	10.9	-8.0
	$Pd(PH_3)$	-13.7	21.6	14.3
	$Pd(PH_3)_2$	0.3	44.6	27.2
	$Pd(PH_2C_2H_4PH_2)$	-4.0	31.1	1.4

[a] Computed at ZORA-BLYP/TZ2P

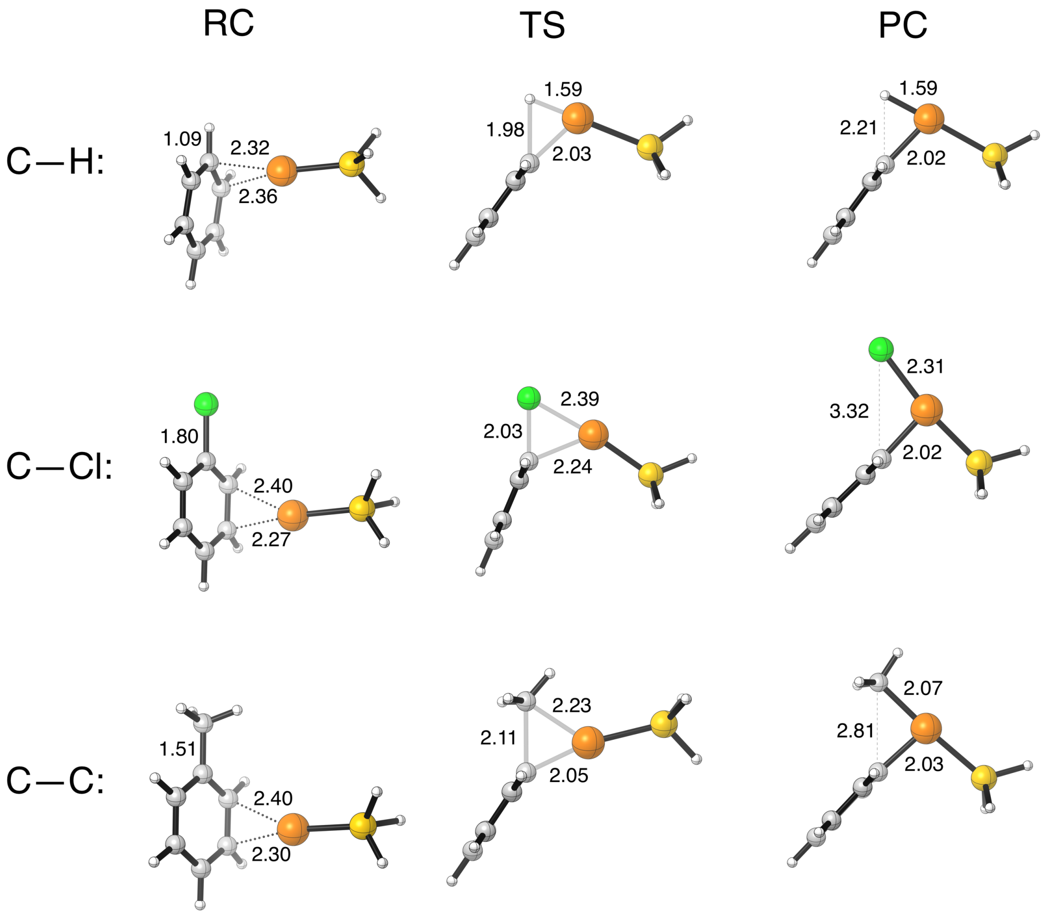

Supplementary Figure 1. Representative geometries of the stationary points (in Å) for the oxidative insertion of Pd(PH_3) into the C–H, C–Cl, and C–C bond, computed at ZORA-BLYP/TZ2P.

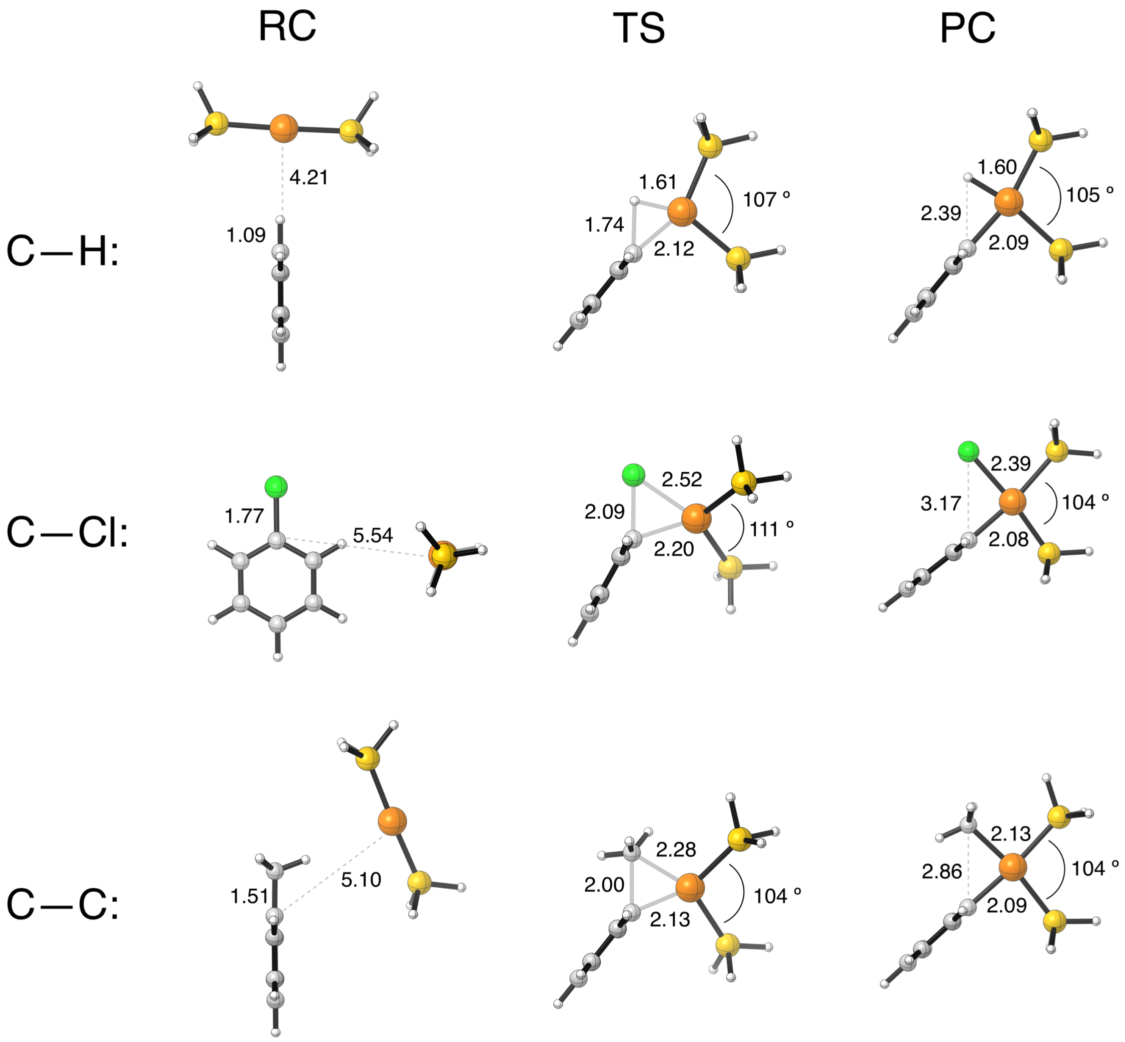

Supplementary Figure 2. Representative geometries of the stationary points (in Å) for the oxidative insertion of $Pd(PH_3)_2$ into the C–H, C–Cl, and C–C bond, computed at ZORA-BLYP/TZ2P (for all RC: dashed line = Pd–C distance).

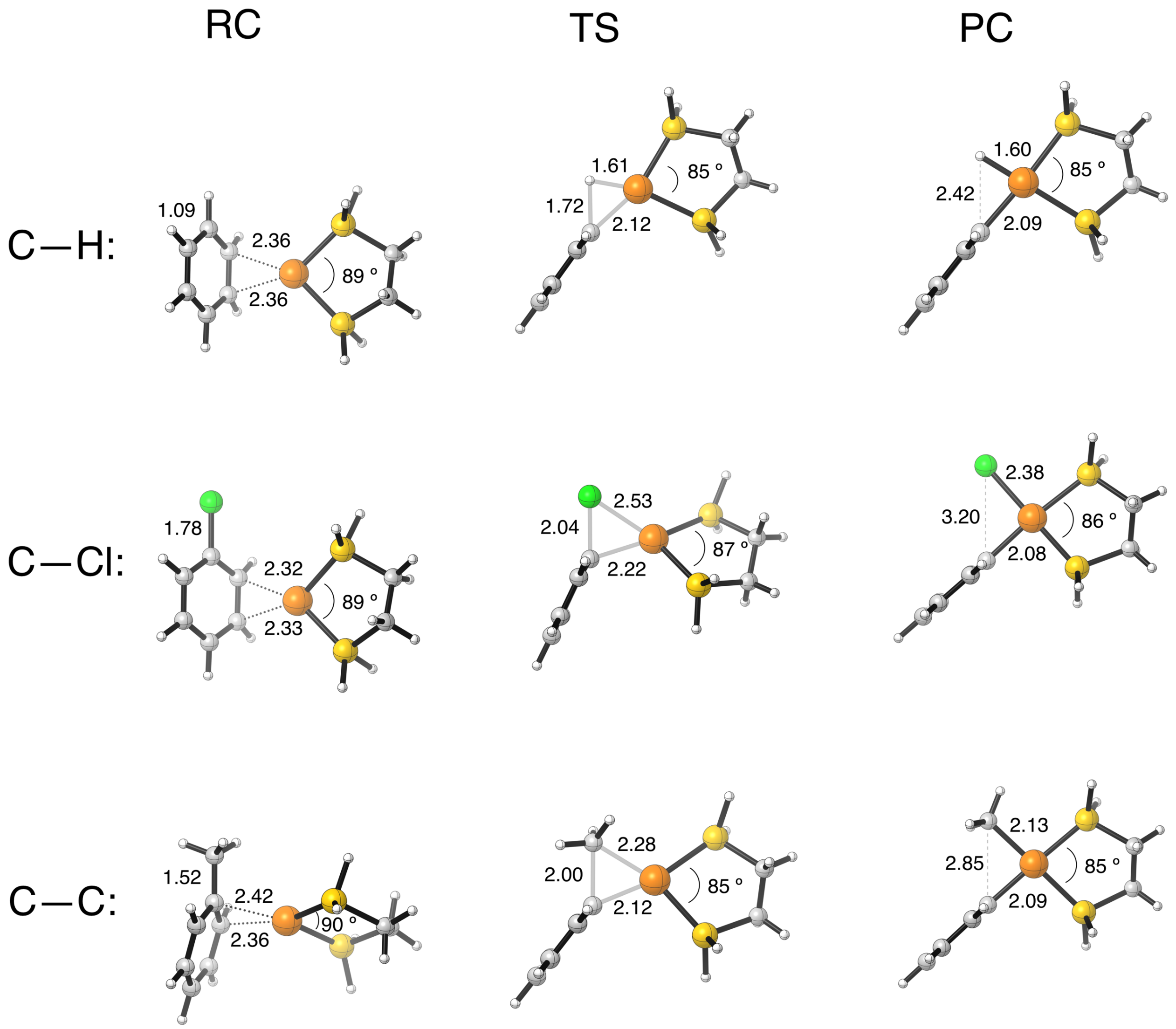

Supplementary Figure 3. Representative geometries of the stationary points (in Å) for the oxidative insertion of Pd($PH_2C_2H_4PH_2$) into the C–H, C–Cl, and C–C bond, computed at ZORA-BLYP/TZ2P.

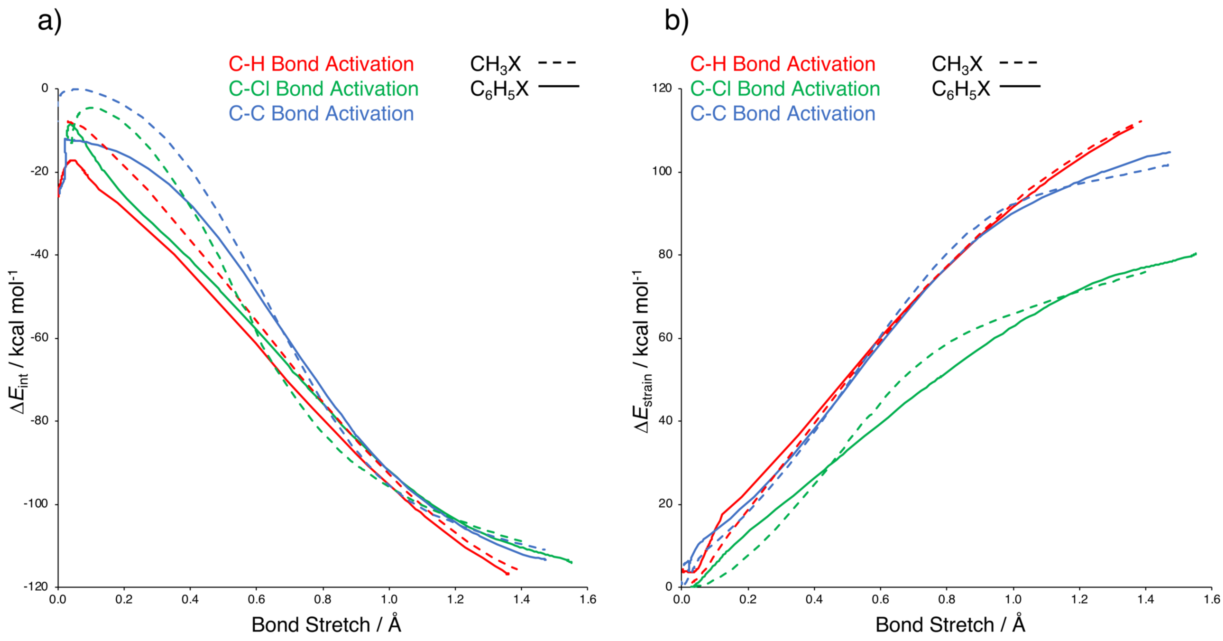

Supplementary Figure 4. Zoomed-in activation strain diagram of a) the strain energies and b) the interaction energies comparing the oxidative insertion of the aliphatic C–X bond versus the arylic C–X bond (X = H, Cl, CH_3) by a Pd catalyst.

Supplementary Table 2. Cartesian coordinates (in Å) and ADF total bonding energies (in kcal mol^{-1}) of all molecular species in this study, computed at ZORA-BLYP/TZ2P.

**C_6H_6**			**[-1666.22]**
C	0.000000	0.000000	0.143795
C	0.000000	1.212661	-0.556419
C	0.000000	1.212661	-1.956702
C	0.000000	0.000000	-2.656917
C	0.000000	-1.212661	-1.956702
C	0.000000	-1.212661	-0.556419
H	0.000000	2.155100	-0.012392
H	0.000000	2.155099	-2.500733
H	0.000000	0.000000	-3.745193
H	0.000000	-2.155099	-2.500733
H	0.000000	-2.155100	-0.012392
H	0.000000	0.000000	1.232070

**C_6H_5Cl**			**[-1626.09]**
C	0.000000	0.000000	-0.242718
C	0.000000	1.218777	-0.926789
C	0.000000	1.210300	-2.326521
C	0.000000	0.000000	-3.029266
C	0.000000	-1.210300	-2.326521
C	0.000000	-1.218777	-0.926789
H	0.000000	2.153879	-0.374170
H	0.000000	2.155766	-2.864642
H	0.000000	0.000000	-4.116773
H	0.000000	-2.155766	-2.864642
H	0.000000	-2.153879	-0.374170
Cl	0.000000	0.000000	1.530755

**$C_6H_5CH_3$**			**[-2030.53]**
C	-1.228226	0.829492	-0.033507
C	-2.434553	0.109475	-0.071390
C	-2.441196	-1.289409	-0.045441
C	-1.235910	-1.997692	0.017513
C	-0.026747	-1.294222	0.052233
C	-0.025739	0.104818	0.026017
H	-3.378916	0.649894	-0.124648
H	-3.387742	-1.825634	-0.078204
H	-1.238786	-3.085518	0.034643
H	0.917240	-1.834206	0.095824
H	0.921949	0.641442	0.049194
C	-1.225883	2.346111	-0.030474
H	-0.311102	2.745753	-0.482841
H	-1.282007	2.737517	0.995349
H	-2.084044	2.748698	-0.580681

Pd ($d^{10}s^0$) **[0.00]**

**Pd(PH_3)**			**[-382.57]**
Pd	0.000000	0.000000	-1.109814
P	0.000000	0.000000	1.065827
H	-0.611742	-1.059568	1.800681
H	1.223484	0.000000	1.800681
H	-0.611742	1.059568	1.800681

**Pd(PH_3)$_2$**			**[-754.24]**
Pd	-2.441975	2.361091	0.000259
P	-0.760661	3.853019	0.412772
H	-0.257945	4.051999	1.732433
H	0.517080	3.712934	-0.204962
H	-0.912293	5.238301	0.109730
P	-4.120924	0.866507	-0.412755
H	-4.719477	0.119036	0.643984
H	-5.349774	1.281338	-1.005637
H	-3.917430	-0.249550	-1.276962

**Pd($PH_2C_2H_4PH_2$)**			**[-1297.60]**
Pd	-0.810546	-1.342404	-0.178152
P	0.801185	0.257050	-0.369941
C	-0.218989	1.760110	0.209723
C	-1.701812	1.712102	-0.233869
P	-2.568749	0.063781	0.175341
H	0.248727	2.687677	-0.141348
H	-0.153322	1.755137	1.305522
H	-1.772459	1.818832	-1.324154
H	-2.255063	2.546926	0.212707
H	2.018413	0.602851	0.307682
H	1.238430	0.757607	-1.639312
H	-3.046252	0.380362	1.488628
H	-3.817061	0.360607	-0.467947

**RC: Pd + C_6H_6**			**[-1687.91]**
C	-0.341105	-0.080778	1.821718
C	-1.627986	0.377569	1.368555
C	-2.532017	-0.561162	0.790405
C	-2.166403	-1.894589	0.641419
C	-0.903739	-2.344706	1.086992
C	-0.009366	-1.460214	1.679992
H	-1.810093	1.442753	1.229365
H	-3.497456	-0.213530	0.428526
H	-2.854830	-2.596093	0.174606
H	-0.628529	-3.389745	0.960529
H	0.970174	-1.805584	2.004031
H	0.453255	0.636683	2.025276
Pd	-1.652309	0.208961	3.564508

**TS: Pd + C_6H_6**			**[-1667.83]**
C	0.092395	-0.577941	-0.209612
C	-1.145418	-1.250158	-0.153715
C	-1.182596	-2.637364	0.033735
C	0.005580	-3.370740	0.135089
C	1.237079	-2.711825	0.039160
C	1.286217	-1.325058	-0.148419
H	-2.074209	-0.694654	-0.251062
H	-2.144113	-3.144759	0.091608
H	-0.027919	-4.449458	0.273564
H	2.165274	-3.277354	0.101500
H	2.247907	-0.827890	-0.241578
Pd	0.154496	1.421045	0.044451
H	0.132305	0.665364	-1.344002

**PC: Pd + C_6H_6**			**[-1672.22]**
C	0.092694	-0.564821	-0.106681
C	-1.146774	-1.226357	-0.080895
C	-1.182925	-2.624427	0.023104
C	0.005110	-3.362164	0.076427
C	1.236908	-2.700176	0.020662
C	1.288354	-1.302628	-0.083481
H	-2.076439	-0.666499	-0.138606
H	-2.145062	-3.133038	0.053974
H	-0.028750	-4.447230	0.149682
H	2.165335	-3.268046	0.049661
H	2.251219	-0.802169	-0.143217
Pd	0.154273	1.402595	0.028809
H	0.155094	1.425426	-1.518418

RC: Pd + C_6H_5Cl [-1637.74]

C	-0.349156	-1.297321	-0.175727
C	-1.579926	-1.897800	0.091467
C	-1.593747	-3.221966	0.545884
C	-0.395151	-3.922606	0.725236
C	0.826759	-3.296923	0.449373
C	0.859344	-1.973848	-0.006064
H	-2.504117	-1.345731	-0.054888
H	-2.546303	-3.702986	0.757691
H	-0.412975	-4.951446	1.076908
H	1.761425	-3.836462	0.587008
H	1.801175	-1.480073	-0.228306
Cl	-0.321816	0.421194	-0.725292
Pd	-0.155686	0.767376	-3.035138

TS: Pd + C_6H_5Cl [-1634.14]

C	-0.951689	0.071017	0.775698
C	-2.312597	-0.256241	0.716082
C	-2.671761	-1.562686	0.367578
C	-1.691827	-2.516157	0.063235
C	-0.338452	-2.157470	0.103401
C	0.045377	-0.857243	0.448994
H	-3.066962	0.490788	0.943112
H	-3.725724	-1.830073	0.330579
H	-1.980753	-3.527750	-0.211358
H	0.429106	-2.888996	-0.140208
H	1.091922	-0.569120	0.471568
Cl	-0.478054	1.801573	1.062011
Pd	-0.460577	0.894043	3.302567

PC: Pd + C_6H_5Cl [-1659.86]

C	0.019635	0.509587	0.153100
C	-1.162989	-0.134413	-0.230665
C	-1.112345	-1.509541	-0.512801
C	0.083912	-2.217364	-0.356156
C	1.239877	-1.559984	0.083535
C	1.214667	-0.185774	0.362897
H	-2.104893	0.404331	-0.310260
H	-2.018463	-2.021160	-0.831225
H	0.110861	-3.286367	-0.555757
H	2.166799	-2.113486	0.220940
H	2.104012	0.322562	0.723854
Pd	-0.032386	2.452562	0.097008
Cl	0.334842	3.060468	2.248507

RC: Pd + $C_6H_5CH_3$ [-2050.26]

C	-1.762009	-0.580511	0.500038
C	-2.955591	-1.350710	0.316353
C	-2.920985	-2.738320	0.226140
C	-1.701736	-3.436643	0.357735
C	-0.526029	-2.736432	0.604073
C	-0.528586	-1.311686	0.678995
H	-3.898628	-0.822650	0.181973
H	-3.843115	-3.288619	0.050276
H	-1.684195	-4.521085	0.274900
H	0.419426	-3.266121	0.701373
H	0.421569	-0.782317	0.603043
C	-1.757755	0.889732	0.101683
H	-0.869018	1.410405	0.470701
H	-2.643578	1.409167	0.484768
H	-1.770867	0.982419	-0.995102
Pd	-1.286842	-0.766066	2.663645

TS: Pd + $C_6H_5CH_3$ [-2019.51]

C	-1.161243	1.445547	-0.508389
C	-2.453237	0.871408	-0.507934
C	-2.639769	-0.456434	-0.108580
C	-1.545725	-1.243806	0.271676
C	-0.258052	-0.693219	0.248947
C	-0.061122	0.633911	-0.147722
H	-3.309441	1.462014	-0.825003
H	-3.643803	-0.877662	-0.101260
H	-1.693755	-2.279111	0.571130
H	0.599601	-1.300034	0.534798
H	0.947374	1.039187	-0.183927
Pd	-1.066770	3.386284	0.077980
C	-0.804780	2.732001	-2.000546
H	-0.684992	3.832345	-2.079240
H	-1.653041	2.428823	-2.611110
H	0.114899	2.256550	-2.336032

PC: Pd + $C_6H_5CH_3$ [-2039.07]

C	-1.230668	1.375719	-0.085123
C	-2.488862	0.787854	-0.301997
C	-2.641645	-0.599623	-0.167992
C	-1.545524	-1.405087	0.162013
C	-0.289208	-0.820515	0.356367
C	-0.123293	0.566193	0.223806
H	-3.347625	1.398602	-0.568137
H	-3.621074	-1.047698	-0.327832
H	-1.667964	-2.481923	0.258416
H	0.570979	-1.440579	0.603749
H	0.862591	1.002929	0.362506
Pd	-1.029489	3.341523	-0.026831
C	-0.893100	3.616289	-2.038074
H	-0.749158	4.703552	-2.122378
H	-1.821597	3.302387	-2.521218
H	-0.033325	3.072895	-2.436639

RC: $Pd(PH_3)$ + C_6H_6 [-2063.71]

C	-0.110812	-0.161665	2.387936
C	-0.283685	0.644183	1.263317
C	-1.019243	0.173669	0.160053
C	-1.576069	-1.104436	0.180769
C	-1.381237	-1.950891	1.300493
C	-0.645211	-1.471917	2.416373
H	0.148883	1.642153	1.239597
H	-1.154305	0.809613	-0.712198
H	-2.136282	-1.472622	-0.675603
H	-1.666346	-2.999630	1.240255
H	-0.372959	-2.147588	3.224181
H	0.459650	0.201022	3.239987
Pd	-2.949923	-1.320836	2.885482
P	-4.803377	-1.073493	4.114530
H	-5.228828	-2.098331	5.012517
H	-4.950040	-0.006592	5.051912
H	-6.083433	-0.888575	3.509309

TS: $Pd(PH_3)$ + C_6H_6 [-2031.73]

C	0.104557	-0.538620	-0.238077
C	-1.145457	-1.173333	-0.167430
C	-1.215542	-2.552197	0.079915
C	-0.047264	-3.308645	0.224324
C	1.197313	-2.678956	0.112777
C	1.278791	-1.300708	-0.134315
H	-2.062774	-0.604563	-0.294651
H	-2.190525	-3.032352	0.149375
H	-0.106017	-4.379432	0.408318
H	2.114328	-3.258513	0.208183
H	2.253840	-0.831440	-0.235540
Pd	0.211228	1.490488	-0.209860
H	0.193843	0.835971	-1.660394
P	0.236520	2.473762	1.980386
H	-0.842499	2.234109	2.878272
H	1.278275	2.149177	2.895429
H	0.291425	3.884316	2.189445

PC: $Pd(PH_3)$ + C_6H_6 [-2031.92]

C	0.107181	-0.489488	-0.174339
C	-1.143560	-1.117933	-0.087596
C	-1.214497	-2.497192	0.161008
C	-0.046817	-3.255495	0.298474
C	1.198145	-2.628189	0.180309
C	1.280951	-1.249621	-0.068148
H	-2.061116	-0.548051	-0.208781
H	-2.190115	-2.975074	0.237132

H	-0.106428	-4.325943	0.484082
H	2.114870	-3.208896	0.271486
H	2.256668	-0.782644	-0.174300
Pd	0.216354	1.526344	-0.276779
H	0.210315	1.021721	-1.784554
P	0.217511	2.246022	2.026018
H	-0.859622	1.863510	2.875361
H	1.262471	1.821805	2.895848
H	0.241170	3.617024	2.421836

RC: Pd(PH_3)$_2$ + C_6H_6 [-2420.86]

C	-1.402965	-2.053906	-0.102300
C	-0.311923	-2.921878	0.029462
C	0.113279	-3.686189	-1.064155
C	-0.552531	-3.582718	-2.291708
C	-1.643739	-2.715224	-2.424547
C	-2.068242	-1.951414	-1.330290
H	0.205625	-3.001818	0.983586
H	0.961175	-4.360635	-0.960379
H	-0.222213	-4.176132	-3.142059
H	-2.161583	-2.634664	-3.378483
H	-2.916080	-1.276810	-1.433366
H	-1.732464	-1.458733	0.749176
H	-5.102965	-1.744623	3.129605
H	-4.812213	-1.013274	5.093128
H	-3.551154	-2.531109	4.331262
Pd	-2.601818	0.352059	3.134298
P	-1.191068	1.995959	2.399089
H	0.214947	1.777073	2.309377
H	-1.329736	2.563895	1.098729
H	-1.099222	3.239667	3.090053
P	-4.028461	-1.250120	3.925978

TS: Pd(PH_3)$_2$ + C_6H_6 [-2393.70]

C	-0.052944	-0.503641	-0.305974
C	-1.200252	-1.284446	-0.065227
C	-1.091261	-2.641683	0.265936
C	0.165316	-3.253931	0.338540
C	1.311587	-2.496874	0.070136
C	1.203115	-1.139585	-0.260960
H	-2.189189	-0.834992	-0.133253
H	-1.992709	-3.221840	0.459013
H	0.249353	-4.309662	0.588597
H	2.295007	-2.963540	0.109826
H	2.107333	-0.575936	-0.482977
Pd	-0.189058	1.609259	-0.410844
H	-0.226036	0.584056	-1.649335
P	-0.038574	2.134927	1.935047
H	-1.005293	1.549580	2.804434
H	1.099780	1.655773	2.647473
H	-0.057035	3.422034	2.559197
P	-0.405167	3.650947	-1.556466
H	-1.560075	3.859974	-2.365247
H	-0.412525	4.958297	-0.976353
H	0.539075	3.965826	-2.576609

PC: Pd(PH_3)$_2$ + C_6H_6 [-2396.21]

C	-0.043980	-0.453085	-0.169976
C	-1.190685	-1.240253	0.045207
C	-1.087080	-2.612565	0.317569
C	0.167577	-3.229695	0.371529
C	1.316186	-2.462595	0.147725
C	1.210096	-1.090362	-0.124328
H	-2.181207	-0.790348	-0.003203
H	-1.990849	-3.198615	0.481229
H	0.248749	-4.295119	0.578667
H	2.299440	-2.930864	0.178340
H	2.120780	-0.521714	-0.306588
Pd	-0.196297	1.605844	-0.495964
H	-0.283759	1.065559	-1.995735
P	-0.025457	1.947511	1.867027
H	-0.984673	1.292101	2.688461
H	1.128847	1.426750	2.515836
H	-0.048604	3.197651	2.558892
P	-0.408490	3.750264	-1.421470
H	-1.560190	4.014321	-2.214323
H	-0.426967	4.989467	-0.709097
H	0.550562	4.149119	-2.394101

RC: Pd($PH_2C_2H_4PH_2$) + C_6H_6 [-2971.49]

Pd	2.429804	0.318515	0.397763
P	1.203361	2.216119	-0.200194
C	-0.572086	1.587341	-0.458103
C	-0.600023	0.165469	-1.058920
P	0.589897	-1.011895	-0.157308
H	-1.139333	2.283232	-1.085818
H	-1.037314	1.586346	0.536591
H	-0.262962	0.189289	-2.103742
H	-1.618844	-0.237151	-1.050805
H	0.870911	3.436323	0.475679
H	1.404213	2.845284	-1.471466
H	-0.303701	-1.497558	0.851794
H	0.413125	-2.146600	-1.016325
C	5.494150	1.232876	0.323141
C	4.601645	0.640727	1.261776
C	4.365974	-0.766455	1.203758
C	5.028550	-1.539378	0.207897
C	5.884524	-0.937124	-0.700085
C	6.119541	0.459932	-0.641909
H	4.300480	1.209531	2.138728
H	3.885380	-1.274684	2.036713
H	4.869194	-2.615552	0.179923
H	6.392244	-1.539048	-1.451059
H	6.806078	0.921825	-1.348632
H	5.694344	2.300883	0.384289

TS: Pd($PH_2C_2H_4PH_2$) + C_6H_6 [-2950.21]

C	-1.944662	0.403507	0.102834
C	-3.154766	-0.194114	-0.301070
C	-3.441361	-1.528209	0.016719
C	-2.536994	-2.287044	0.769055
C	-1.346447	-1.694814	1.206747
C	-1.059884	-0.360738	0.889168
H	-3.881383	0.380608	-0.872547
H	-4.378079	-1.971586	-0.318799
H	-2.762762	-3.321224	1.021797
H	-0.639469	-2.269014	1.804388
H	-0.135693	0.082588	1.255282
Pd	-1.329129	2.251655	-0.727675
H	-2.132773	2.018780	0.653621
P	-0.978789	4.541073	-1.104666
C	0.280919	4.758550	-2.504292
C	0.108661	3.658302	-3.574910
P	-0.091697	1.952174	-2.770861
H	1.271047	4.680696	-2.036554
H	0.204351	5.755774	-2.950584
H	0.946839	3.663633	-4.279584
H	-0.809390	3.830872	-4.151781
H	-0.411820	1.188052	-3.934430
H	1.277373	1.547060	-2.712241
H	-2.042787	5.358096	-1.594462
H	-0.473236	5.502913	-0.181175

PC: Pd($PH_2C_2H_4PH_2$) + C_6H_6 [-2953.86]

C	-1.863052	0.391629	-0.042250
C	-3.063743	-0.212208	-0.459589
C	-3.364228	-1.540623	-0.122819
C	-2.472020	-2.293599	0.648803
C	-1.279423	-1.703517	1.081964
C	-0.982074	-0.374798	0.743362
H	-3.783816	0.351790	-1.050932
H	-4.301106	-1.982998	-0.460291
H	-2.706026	-3.322923	0.914648
H	-0.578236	-2.273962	1.690481
H	-0.051627	0.060198	1.105688
Pd	-1.375105	2.333583	-0.632185
H	-2.156433	2.665731	0.723581
P	-0.933620	4.562890	-1.182885

C	0.315879	4.624961	-2.603812
C	0.091116	3.448013	-3.578964
P	-0.173668	1.830862	-2.631458
H	1.307583	4.549914	-2.139468
H	0.268588	5.585500	-3.127844
H	0.923742	3.358002	-4.284557
H	-0.822932	3.609189	-4.164955
H	-0.586553	0.973324	-3.692378
H	1.165563	1.345101	-2.548003
H	-1.980005	5.380510	-1.704130
H	-0.383624	5.544871	-0.310221

RC: Pd(PH$_3$) + C$_6$H$_5$Cl [-2020.59]

C	-0.531676	-1.295112	-0.151231
C	-0.763882	-1.617990	1.185581
C	-0.488207	-2.922967	1.612229
C	0.007355	-3.873318	0.712235
C	0.230173	-3.520211	-0.623572
C	-0.039078	-2.220689	-1.069269
H	-1.148833	-0.873410	1.876688
H	-0.663522	-3.190967	2.651730
H	0.218975	-4.884833	1.050676
H	0.615140	-4.254441	-1.327752
H	0.126504	-1.923745	-2.102634
Cl	-0.886864	0.383430	-0.688670
Pd	-0.427853	0.748374	-3.066559
P	-0.070669	1.238382	-5.183716
H	0.270004	0.222168	-6.127105
H	-1.088158	1.834849	-5.988265
H	0.962276	2.141401	-5.579299

TS: Pd(PH$_3$) + C$_6$H$_5$Cl [-2013.09]

C	0.096303	0.393705	0.675302
C	-1.142452	-0.203505	0.394537
C	-1.154941	-1.458056	-0.225347
C	0.041741	-2.126232	-0.515441
C	1.264885	-1.535536	-0.172529
C	1.305469	-0.282129	0.448415
H	-2.069245	0.305089	0.640523
H	-2.111272	-1.915211	-0.472232
H	0.020606	-3.105731	-0.986964
H	2.200017	-2.052977	-0.378305
H	2.251171	0.166336	0.735777
Pd	0.181646	2.517951	-0.040937
Cl	0.104108	1.717445	2.208993
P	0.255286	3.068173	-2.227752
H	-0.792000	2.659487	-3.104036
H	0.305856	4.419049	-2.685107
H	1.324929	2.600381	-3.045660

PC: Pd(PH$_3$) + C$_6$H$_5$Cl [-2032.50]

C	0.118751	0.443137	-0.163552
C	-1.119130	-0.198963	-0.253653
C	-1.151252	-1.596350	-0.387761
C	0.036721	-2.332750	-0.432633
C	1.265856	-1.672411	-0.338451
C	1.316368	-0.275646	-0.203802
H	-2.049185	0.360731	-0.204482
H	-2.113812	-2.100784	-0.450041
H	0.004760	-3.415290	-0.534263
H	2.196475	-2.236389	-0.362145
H	2.277085	0.224448	-0.115664
Pd	0.175670	2.437094	0.132381
Cl	0.121898	2.521247	2.430988
P	0.235557	2.666334	-2.120465
H	-0.830792	2.150028	-2.906402
H	0.284000	3.973301	-2.683609
H	1.308059	2.092502	-2.856513

RC: Pd(PH$_3$)$_2$ + C$_6$H$_5$Cl [-2380.76]

C	-0.854390	-1.030832	0.917424
C	-0.854133	-2.123900	1.788592
C	-0.518153	-3.387914	1.289648
C	-0.187858	-3.552826	-0.060459
C	-0.194099	-2.445691	-0.916367
C	-0.527615	-1.174751	-0.433451
H	-1.111626	-1.987036	2.835020
H	-0.516053	-4.242830	1.962674
H	0.072372	-4.537409	-0.442185
H	0.059858	-2.559446	-1.968053
H	-0.532531	-0.316334	-1.100584
Cl	-1.280039	0.574514	1.543511
Pd	-0.102982	0.594246	-4.327727
P	-2.315878	0.175704	-4.728024
H	-2.804671	-1.156296	-4.869441
H	-3.324303	0.601995	-3.815061
H	-2.952843	0.686745	-5.896613
P	2.108548	1.041613	-3.955224
H	3.065365	-0.007482	-3.823946
H	2.863223	1.806838	-4.891949
H	2.531650	1.765944	-2.802524

TS: Pd(PH$_3$)$_2$ + C$_6$H$_5$Cl [-2361.14]

C	-0.137384	0.543479	0.470805
C	-1.008661	-0.434109	-0.046367
C	-0.469124	-1.609546	-0.569217
C	0.916985	-1.841951	-0.541655
C	1.762545	-0.890242	0.034976
C	1.242250	0.294084	0.578723
H	-2.081450	-0.263671	-0.053545
H	-1.138282	-2.353216	-0.998784
H	1.324027	-2.767837	-0.940830
H	2.834303	-1.071769	0.093121
H	1.894188	1.002938	1.081083
Pd	-0.255511	2.656137	-0.140905
Cl	-0.911924	1.646906	2.069633
P	-0.927530	2.361092	-2.371938
H	-2.021584	1.506749	-2.716128
H	-1.322243	3.436494	-3.225628
H	-0.021048	1.781330	-3.310506
P	0.566281	4.892361	0.232027
H	0.084964	6.021466	-0.497489
H	0.477891	5.524775	1.510104
H	1.946433	5.200035	0.029547

PC: Pd(PH$_3$)$_2$ + C$_6$H$_5$Cl [-2389.91]

C	-0.005362	0.494405	-0.249009
C	-1.144992	-0.318254	-0.331174
C	-1.015124	-1.715080	-0.357174
C	0.251150	-2.307625	-0.307007
C	1.389015	-1.497814	-0.228077
C	1.263442	-0.100488	-0.202173
H	-2.140036	0.121803	-0.354823
H	-1.908199	-2.335816	-0.409955
H	0.350063	-3.391128	-0.323976
H	2.379298	-1.948226	-0.179758
H	2.160680	0.510538	-0.124906
Pd	-0.201955	2.555867	-0.090462
Cl	-0.355195	2.366113	2.283656
P	-0.036054	2.494194	-2.348899
H	-0.991371	1.730853	-3.070966
H	-0.091802	3.678728	-3.139281
H	1.136505	1.931838	-2.919621
P	-0.443595	4.906656	0.403386
H	-0.476671	5.977845	-0.543958
H	-1.601850	5.271351	1.136482
H	0.530256	5.459889	1.273813

RC: Pd(PH$_2$C$_2$H$_4$PH$_2$) + C$_6$H$_5$Cl [-2932.35]

Pd	2.877776	0.056055	-0.683065
P	1.934395	1.975371	-1.632795
C	0.093116	1.893608	-1.171197
C	-0.439837	0.444234	-1.182504
P	0.715590	-0.742441	-0.253245
H	-0.499308	2.528809	-1.838686
H	0.018351	2.316595	-0.160703

H	-0.491126	0.068587	-2.212982
H	-1.452276	0.402002	-0.766323
H	2.129602	3.375325	-1.397935
H	1.805018	2.112528	-3.052194
H	0.144502	-0.643526	1.055002
H	0.049906	-1.965675	-0.584401
C	5.871048	0.501192	1.640196
C	5.314541	-0.693857	2.166588
C	4.715570	-1.593046	1.306654
C	4.620546	-1.357172	-0.095038
C	5.197156	-0.151044	-0.611462
C	5.820960	0.765983	0.282728
H	5.370301	-0.908007	3.229959
Cl	4.057786	-3.114209	1.943604
H	4.388724	-2.190412	-0.752590
H	5.384957	-0.062609	-1.679119
H	6.277913	1.668354	-0.117426
H	6.354294	1.201516	2.317935

TS: Pd($PH_2C_2H_4PH_2$) + C_6H_5Cl [-2918.19]

C	-2.063323	0.695051	0.497452
C	-3.271007	0.377650	-0.153797
C	-3.504269	-0.951085	-0.540495
C	-2.590982	-1.958354	-0.218798
C	-1.431764	-1.634471	0.510955
C	-1.170908	-0.321270	0.895436
H	-4.010291	1.148988	-0.349987
H	-4.423161	-1.192906	-1.071876
H	-2.787925	-2.989359	-0.502312
H	-0.724654	-2.416248	0.783506
H	-0.280172	-0.073001	1.465398
Pd	-1.365155	2.465533	-0.637061
Cl	-2.193988	2.302221	1.748840
P	-1.442236	4.486496	-1.911635
C	0.125285	4.449798	-2.978213
C	0.444647	3.021910	-3.472925
P	0.374776	1.760719	-2.059800
H	0.935958	4.819910	-2.337085
H	0.032260	5.140123	-3.823281
H	1.420746	2.994040	-3.968917
H	-0.306831	2.698299	-4.204994
H	0.496046	0.565186	-2.835630
H	1.749539	1.784963	-1.657910
H	-2.415644	4.677007	-2.943346
H	-1.394459	5.863029	-1.524732

PC: Pd($PH_2C_2H_4PH_2$) + C_6H_5Cl [-2948.58]

C	-1.784815	0.388833	-0.167693
C	-3.027580	-0.179787	-0.482916
C	-3.301203	-1.514163	-0.147806
C	-2.336628	-2.292743	0.501840
C	-1.096028	-1.729504	0.816836
C	-0.818069	-0.395620	0.478501
H	-3.799562	0.413147	-0.969019
H	-4.274412	-1.939246	-0.389404
H	-2.552088	-3.326681	0.764251
H	-0.339710	-2.322448	1.329324
H	0.150566	0.024439	0.744014
Pd	-1.408539	2.386111	-0.613562
Cl	-2.627214	3.107436	1.296740
P	-0.856302	4.610051	-1.293748
C	0.432930	4.452304	-2.665997
C	0.150366	3.199309	-3.526413
P	-0.261976	1.716749	-2.437070
H	1.403606	4.357348	-2.163053
H	0.469884	5.353376	-3.286983
H	0.994788	2.969474	-4.185857
H	-0.730041	3.361997	-4.161102
H	-0.794727	0.793081	-3.376307
H	1.007753	1.096206	-2.258949
H	-1.838897	5.438736	-1.909065
H	-0.272122	5.605705	-0.462626

RC: Pd(PH_3) + $C_6H_5CH_3$ [-2428.21]

C	-2.138337	-0.344927	-0.008996
C	-3.215366	-1.233101	-0.082249
C	-3.077068	-2.584701	0.284237
C	-1.847905	-3.075219	0.717121
C	-0.725717	-2.210501	0.770517
C	-0.879181	-0.844395	0.420739
H	-4.182157	-0.870273	-0.427837
H	-3.936726	-3.248880	0.224027
H	-1.732554	-4.123402	0.981953
H	0.269324	-2.624572	0.928552
H	-0.000488	-0.206999	0.340358
C	-2.280642	1.107915	-0.413467
H	-2.029299	1.776622	0.419262
H	-3.303271	1.333751	-0.733193
H	-1.604487	1.355005	-1.243189
Pd	-0.865057	-1.149205	2.800458
P	-0.774842	-0.441528	4.917213
H	-1.079595	-1.314014	6.006553
H	0.443850	0.045855	5.479368
H	-1.579075	0.638416	5.393129

TS: Pd(PH_3) + $C_6H_5CH_3$ [-2390.31]

C	-1.236787	1.300065	-0.486036
C	-2.532731	0.764345	-0.350017
C	-2.716996	-0.619596	-0.238918
C	-1.618528	-1.485818	-0.294151
C	-0.330776	-0.961766	-0.462569
C	-0.139006	0.420473	-0.575715
H	-3.397068	1.424128	-0.325461
H	-3.722247	-1.019822	-0.117218
H	-1.765831	-2.561234	-0.219943
H	0.527524	-1.629643	-0.515594
H	0.863885	0.812240	-0.729929
Pd	-0.880014	3.108485	0.408523
C	-1.148209	2.957034	-1.795720
H	-0.885898	4.022730	-1.764915
H	-2.168847	2.826264	-2.150201
H	-0.441752	2.420242	-2.424996
P	-0.490462	4.898974	1.825605
H	-1.413908	5.977593	1.960271
H	-0.356863	4.660292	3.226540
H	0.669172	5.722512	1.718528

PC: Pd(PH_3) + $C_6H_5CH_3$ [-2398.48]

C	-1.180064	1.396382	-0.045431
C	-2.475937	0.875306	0.086061
C	-2.665635	-0.511408	0.191444
C	-1.572080	-1.382929	0.150695
C	-0.282889	-0.860956	0.000038
C	-0.082893	0.524242	-0.105971
H	-3.340275	1.534368	0.107711
H	-3.675616	-0.904576	0.298136
H	-1.723631	-2.457608	0.227056
H	0.576569	-1.528476	-0.043085
H	0.926030	0.908323	-0.234353
Pd	-0.883429	3.400270	-0.038252
C	-1.093493	3.304392	-2.099987
H	-0.943222	4.362473	-2.374974
H	-2.090448	2.974887	-2.406583
H	-0.331829	2.681543	-2.577498
P	-0.617939	3.654871	2.318143
H	-1.674099	3.266671	3.192675
H	0.409824	2.931063	2.989458
H	-0.354572	4.907756	2.950949

RC: Pd($(PH_3)_2$) + $C_6H_5CH_3$ [-2785.04]

C	-1.847926	-2.632183	-0.076977
C	-0.538174	-3.099023	0.128854
C	0.452540	-2.924065	-0.843660
C	0.150977	-2.276702	-2.047201
C	-1.149337	-1.808881	-2.267945
C	-2.136244	-1.986863	-1.292124
H	-0.292624	-3.607983	1.060186
H	1.458853	-3.297398	-0.662737

H	0.918925	-2.143230	-2.806250
H	-1.397563	-1.309078	-3.202543
H	-3.146222	-1.623034	-1.477210
C	-2.910918	-2.794514	0.990840
H	-2.919988	-1.931287	1.674207
H	-2.733310	-3.689861	1.597920
H	-3.911382	-2.872042	0.549691
Pd	-3.087542	0.497442	3.761824
P	-1.619578	1.706520	2.487129
H	-0.328262	1.198445	2.161677
H	-1.951030	2.115768	1.162455
H	-1.167273	2.985521	2.925079
P	-4.550911	-0.640573	5.096443
H	-4.212689	-1.908294	5.655875
H	-5.021079	-0.069866	6.315541
H	-5.836185	-1.036678	4.621454

TS: Pd(PH_3)$_2$ + $C_6H_5CH_3$ [-2739.07]

C	-1.119174	1.432043	-0.399715
C	-2.510607	1.378146	-0.157713
C	-3.119054	0.197483	0.289040
C	-2.365875	-0.966363	0.474207
C	-0.987856	-0.934711	0.208003
C	-0.373236	0.239597	-0.230235
H	-3.124060	2.262354	-0.318977
H	-4.191106	0.189713	0.481613
H	-2.841682	-1.885318	0.809117
H	-0.387706	-1.833897	0.342036
H	0.694242	0.234160	-0.443682
Pd	-0.228902	3.323548	-0.018404
C	-0.720193	2.467566	-2.074934
H	-1.590373	3.058386	-2.367969
H	-0.764455	1.496442	-2.561579
H	0.214946	2.948422	-2.391979
P	0.356502	3.023741	2.271664
H	0.942342	1.783253	2.670236
H	1.223297	3.837111	3.070645
H	-0.693004	2.983738	3.239849
P	0.167739	5.654225	-0.433110
H	1.354894	6.343123	-0.031916
H	0.156010	6.211704	-1.752424
H	-0.721497	6.629395	0.114619

PC: Pd(PH_3)$_2$ + $C_6H_5CH_3$ [-2757.63]

C	-1.036830	1.374646	0.031786
C	-2.403805	1.097901	0.223938
C	-2.870398	-0.221941	0.318216
C	-1.978490	-1.295680	0.219730
C	-0.617049	-1.037316	0.025381
C	-0.152928	0.283539	-0.068592
H	-3.123622	1.912369	0.293656
H	-3.933948	-0.407920	0.464038
H	-2.340154	-2.319855	0.288812
H	0.088479	-1.863286	-0.058618
H	0.910761	0.452632	-0.230816
Pd	-0.343899	3.342710	-0.075626
C	-0.698785	3.169519	-2.168264
H	-1.457562	3.914142	-2.438442
H	-1.044967	2.173889	-2.444496
H	0.238807	3.394137	-2.691304
P	-0.067669	3.189864	2.282009
H	-1.216434	2.851842	3.050641
H	0.769378	2.148663	2.771961
H	0.416484	4.207699	3.162886
P	0.398160	5.563683	-0.421005
H	1.750059	5.938743	-0.160164
H	0.325565	6.126221	-1.727440
H	-0.190919	6.668670	0.263458

RC: Pd($PH_2C_2H_4PH_2$) + $C_6H_5CH_3$ [-3333.42]

Pd	2.093483	0.445843	-0.062047
P	2.506540	2.309390	-1.411165
C	1.926501	1.777745	-3.142345
C	2.207788	0.284188	-3.414221
P	1.652771	-0.820375	-1.969534
H	2.390173	2.404086	-3.912308
H	0.845555	1.969386	-3.168248
H	3.287512	0.116232	-3.522267
H	1.729904	-0.035179	-4.346837
H	1.985891	3.645650	-1.449464
H	3.844129	2.705823	-1.740002
H	0.309744	-1.079628	-2.397068
H	2.168821	-2.058865	-2.477008
C	3.588012	0.994056	2.636949
C	2.235188	0.734106	2.278083
C	1.788161	-0.612988	2.096606
C	2.751864	-1.653927	2.259025
C	4.068125	-1.380358	2.595650
C	4.492532	-0.044169	2.792315
H	1.503308	1.534344	2.374385
H	-0.022365	-1.129388	3.164354
H	2.427168	-2.686581	2.137744
H	4.776273	-2.197298	2.719384
H	5.522567	0.163948	3.073868
H	3.898955	2.023365	2.805153
C	0.303887	-0.955447	2.127204
H	-0.311730	-0.147722	1.718156
H	0.092796	-1.866694	1.557240

TS: Pd($PH_2C_2H_4PH_2$) + $C_6H_5CH_3$ [-3296.44]

C	-2.027622	0.623479	0.343675
C	-3.222152	0.082278	-0.184284
C	-3.438569	-1.301341	-0.203519
C	-2.488887	-2.179777	0.329732
C	-1.313749	-1.652463	0.888481
C	-1.088691	-0.275127	0.907562
H	-3.986280	0.744474	-0.586885
H	-4.361359	-1.690972	-0.631784
H	-2.663014	-3.253443	0.322714
H	-0.566645	-2.321729	1.313671
H	-0.174787	0.109027	1.357176
Pd	-1.333356	2.391584	-0.606710
C	-2.381883	2.269593	1.417045
P	-1.089543	4.640452	-1.415000
C	0.236034	4.603325	-2.771150
C	0.144332	3.308172	-3.607234
P	-0.015206	1.785687	-2.488683
H	1.201901	4.651821	-2.251215
H	0.161497	5.488073	-3.411899
H	1.005874	3.215659	-4.276888
H	-0.758918	3.323722	-4.231102
H	-0.261394	0.801784	-3.497535
H	1.371488	1.463972	-2.335374
H	-2.156159	5.238964	-2.158368
H	-0.690831	5.859403	-0.776483
H	-2.527966	1.577614	2.242394
H	-3.339955	2.726320	1.158449
H	-1.656816	3.025459	1.746321

PC: Pd($PH_2C_2H_4PH_2$) + $C_6H_5CH_3$ [-3316.13]

C	-1.859794	0.438525	-0.055002
C	-3.050652	-0.181053	-0.477800
C	-3.335746	-1.513759	-0.143789
C	-2.433663	-2.258327	0.624477
C	-1.246092	-1.656672	1.055288
C	-0.964467	-0.323743	0.718740
H	-3.777372	0.374442	-1.069573
H	-4.267324	-1.966621	-0.482112
H	-2.655121	-3.291119	0.887435
H	-0.536008	-2.221681	1.658612
H	-0.035353	0.118404	1.076673
Pd	-1.416170	2.408954	-0.595217
C	-2.462925	2.935475	1.185691
P	-0.900322	4.606041	-1.260471
C	0.388288	4.550402	-2.643073
C	0.137630	3.336334	-3.565321
P	-0.224290	1.790506	-2.536505
H	1.362591	4.456389	-2.146437
H	0.396500	5.485760	-3.212602

H	0.983281	3.173817	-4.242045	H	-0.349421	5.625370	-0.429008
H	-0.753026	3.506690	-4.183758	H	-1.960628	2.455998	2.031486
H	-0.681401	0.899266	-3.550432	H	-3.484109	2.549466	1.110488
H	1.083686	1.232391	-2.413818	H	-2.486480	4.019599	1.335680
H	-1.905549	5.423540	-1.860630				
